# Quantifying the Pathway and Predicting Spontaneous Emulsification during Material Exchange in a Two Phase Liquid System

**DOI:** 10.1038/s41598-017-14638-9

**Published:** 2017-10-30

**Authors:** Stephen Spooner, Alireza Rahnama, Jason M. Warnett, Mark A. Williams, Zushu Li, Seetharaman Sridhar

**Affiliations:** 1 WMG, University of Warwick, Coventry, CV4 7AL UK; 2 Colorado School of Mines, George S. Ansell, Department of Metallurgical and Materials Engineering, Golden, CO 80401 USA

## Abstract

Kinetic restriction of a thermodynamically favourable equilibrium is a common theme in materials processing. The interfacial instability in systems where rate of material exchange is far greater than the mass transfer through respective bulk phases is of specific interest when tracking the transient interfacial area, a parameter integral to short processing times for productivity streamlining in all manufacturing where interfacial reaction occurs. This is even more pertinent in high-temperature systems for energy and cost savings. Here the quantified physical pathway of interfacial area change due to material exchange in liquid metal-molten oxide systems is presented. In addition the predicted growth regime and emulsification behaviour in relation to interfacial tension as modelled using phase-field methodology is shown. The observed *in-situ* emulsification behaviour links quantitatively the geometry of perturbations as a validation method for the development of simulating the phenomena. Thus a method is presented to both predict and engineer the formation of micro emulsions to a desired specification.

## Introduction

Reaction rates dependent on the interfacial area between two phases is an encompassing multidisciplinary expanse of study applicable to problems ranging from biological systems to refining of metals and geological situations. The problem approach is imperative, as when diffusion in the bulk is rate limiting, the reduction in diffusion length offered by mixing of systems can drastically speed up the rate of a reaction. This results in higher efficiency and, if the necessary magnitude of change occurs, a different rate limiting step. This can often manifest as spontaneous emulsification (SE)^1,2^ of the species to form momentarily stabilized micro pockets of one phase dispersed through the other^3,4^. Considering this, it is desirable to create a system with low interfacial tension, to allow the maximum sustained interfacial area. This can be aided or stabilized^5^ through several mechanisms: bi-layer formation, induced charge gradients, surfactant addition and material exchange^6–9^.

In addition to classical areas^10–12^ where interfacial breakdown is sought to reduce or remove a kinetic barrier or naturally occurring high-temperature reactions in geochemical systems, known systems where SE occurs can be utilized to produce desirable micro dispersions instead of traditional physical sheer methods^13^. Examples include silicon particle production^14^, controlling the morphology of polymeric membranes^15^ and even the formation of edible systems for controlled taste without effecting texture^16^. Interfacial breakdown (or the avoidance thereof) is critical for manufacturing of metals which are more often than not processed in molten state wherein the molten metal is in contact with a molten oxide phase which serves to protect the metal from the environment and absorb impurities^17^. This last case is especially difficult to study since molten metals are processed at temperatures where imaging is difficult and rates are fast.

Mapping the transient interfacial area is key to understanding the rate of material exchange between two immiscible liquids, as well as controlling and predicting the phenomena such as the “tears of wine” or soap-aided spreading between hydrocarbons and water^18^. These are prime examples of how interfaces can behave in the presence of surface active species and physical influences causing a favourable increase in immiscible phase contact area. The authors hypothesise that the manifestations of these physical phenomena are controlled through the systematic growth, necking and finally budding (when SE is considered) of perturbations at the liquid-liquid interface. This cycle follows strict geometries controlled by the fundamental properties of the species such that the resultant geometries can be understood and predicted mathematically.

Here two high-temperature systems, of relevance to metals manufacturing, are explored where the interfacial exchange of material is thermodynamically favourable and very fast in comparison to the mass transport of material in their bulk phases^19^. This situation is unusual in the lower temperature systems home to classical interface studies, where the reaction at the interface is often rate limiting^20^. The systems being investigated consist of two immiscible phases, a molten metal and a molten oxide. Such systems are critical for extraction refining, casting and welding of metallic alloys. System 1 consists of a high-purity iron droplet and a high level of iron(ll) oxide in the molten oxide phase (Fe-FeO system) to allow for quantification of the interface at varying stages of emulsification. Molten oxide phases (also known as slags) are inevitably present during extraction and refining of metals, and are partly a result of the parent minerals and partly engineered to promote various processes. The active reaction within this system is displayed in equation 1, and is of practical importance in the conversion of iron to steel during integrated steel manufacturing. System 2 uses a high-aluminium alloyed iron droplet and an enriched silicon dioxide oxide phase (FeAl-SiO_2_ system) for *in-situ* observation of the emulsification’s physical pathway. The active reaction is shown in equation 2. The latter is relevant for processing of low-density advanced high-strength steels for automotive components, but also has the convenience of study in the present discussion due to lack of transition metals in the oxide phase, making this phase optically transparent. The system has previously been studied by the present authors to quantify the driving force required for emulsification by changing the chemical potential across the interface through differing starting compositions (0 to 8% Al)^21^. The study showed free energy stabilization was enough to drive the phenomena against the cost of increased interfacial tension. However no quantification of the pathway of spontaneous emulsification has been possible previously.

1 $$(FeO)\to [Fe]+[O]$$

2 $$4[Al]+3(Si{O}_{2})\to 3[Si]+2(A{l}_{2}{O}_{3})$$

where species in [] are present in the liquid metal phase, and those in () are within the molten oxide phase.

The findings from these experiments and further input from the literature have been used to create a phase-field representation of the systems, where emulsification behaviour can be tracked and predicted through both chemical gradient mapping and graphical representation of the interface morphology. It is suggested while reaction is occurring in these systems there is a chance the interface between the two phases perturbs. In addition, depending on the balance between interfacial tension and chemical driving force the system may break up into an emulsion to facilitate the faster dissipation of free energy.

## Experimental

### Materials

All molten oxide mixtures were prepared through powder mixing of reagent grade: CaO, MgO, SiO_2_, Al_2_O_3_, CaO•P2O_5_, and FeO followed by pre-melting at 1873 K for 2 hours in a horizontal tube furnace with use of either MgO (Fe-FeO system) or Al_2_O_3_ crucibles (FeAl-SiO_2_ and null system). FeO was prepared through heating of Fe_2_O_3_ and Fe at 1273 K for 4 hours in a Fe crucible using a 99.9999% Ar environment (used for the mixture pre-melts as well). After pre-melting the oxide mixtures were then ground using a disk mill. Oxide mixture compositions were measured using X-ray fluoroscopy (XRF) by West Penn Testing.

Samples containing the iron alloy of composition 0.0003% Mn, 0.0004% P, 0.0001% Ni, 0.0003% Cr, 0.0005% Al, 0.004% C, 0.001% S, 0.0034% O, 0.001 N, and slag of composition 36.89% CaO, 7.14% MgO, 16.21% SiO_2_, 32.31% FeO_t_, 1.65% P_2_O_5_, comprise the Fe-FeO system. Experiments were conducted using high-density MgO crucibles.

Samples containing the iron alloy of the same composition as the Fe-FeO system and slag with the composition 43.02% CaO, 8.74% MgO, 24.11% SiO_2_, 24.11% Al_2_O_3_ comprise the null experimental system. Experiments were conducted using high-density MgO crucibles.

Samples containing the iron alloy of composition 0.0002% Mn, 0.0004% P, 0.002% Ni, 0.0005% Cr, 7.87% Al, 0.0007% C, 0.001% S, 0.0038% O, 0.001% N and slag of composition 36.17% CaO, 23.11% SiO_2_, 38.53% Al_2_O_3_ comprise the FeAl-SiO_2_ system. Experiments were conducted using sapphire crucibles.

All oxide powders were procured from Sigma-Aldrich and metal alloys made in-house using stock Ferro alloys and electrically made “pure” iron procured from Alfa Aesar. Alloy compositions were tested using inductively coupled plasma mass spectroscopy with the addition of LECO analysis for O, C and N.

### High-Temperature Confocal Scanning Laser Microscopy

The high-temperature confocal scanning laser microscope (HT-CSLM) (Yonekura VL2000DX-SVF17SP) consists of an elliptical gold-coated chamber, where a halogen bulb is situated in the lower focal point, and the sample in the other. In this way infrared (IR) radiation produced from the bulb is reflected and focused onto the sample for heating. The sample stage consists of a protruding alumina rod (into the centre of the second focal point), where an R-type thermocouple passes through the rod and is attached to a platinum ring at the end, on which the sample is positioned. A scanning UV laser is then used to view the sample while the experiment is conducted, recording at 15fps with use of the HiTOS software package. A widening aperture function (inbuilt within the machine) was also used on the laser focusing to increase field of view in the z-direction (this reduces resolution in grey-scale accuracy when analysing the recorded video, this is the reason size quantification from the HT-CSLM images is not sufficient alone to report droplet feature sizes).

**Figure 1:**
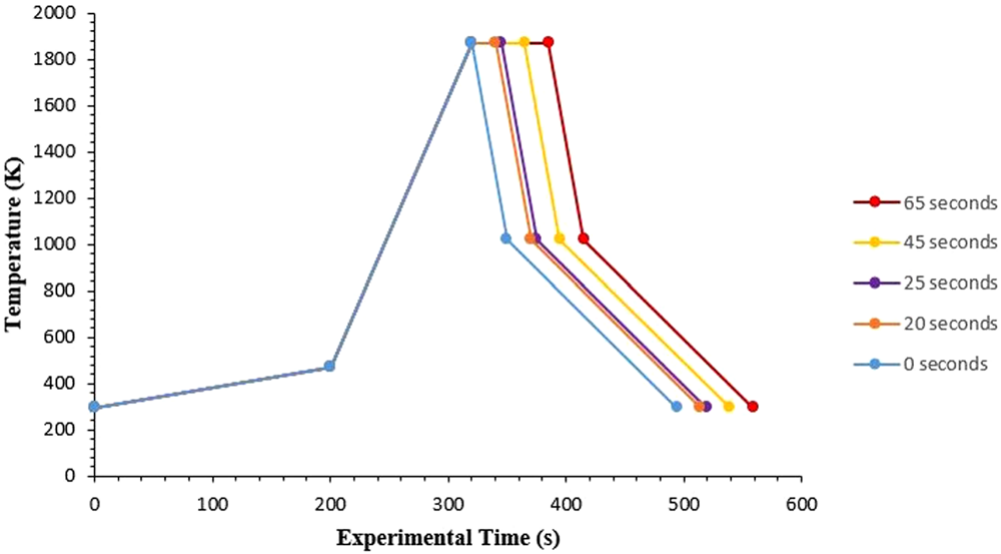
The programmed heating cycle of the HT-CSLM, showing a slow early heating regime and a quench point on cooling for protection of the equipment against thermal shock.

0.2 g (with a range of ±0.034g) of molten oxide was loaded into a crucible. The mix was then pre-melted using the HT-CSLM, following the heating cycle as shown in Fig. 1 with the 0-second hold. After cooling a metal cylinder of dimensions 1.19 mm H, 1.49 mm D weighing 17 mg (with a range of ±0.52) is added into the centre of the quenched molten oxide meniscus and a further 0.3 g (with a range of ±0.12g) of molten oxide is then added on top of the cylinder. This is followed by lightly compressing the powder by hand before the sample is loaded into the HT-CSLM for the experimental heating cycle. In the case of the null experiment system and the FeAl-SiO_2_ system samples, a separate Pt and Al_2_O_3_ spacer are added to the underside of the crucible. The platinum spacer is used to increase reflected light through the sample, giving greater depth of vision in the HT-CSLM. The alumina spacer is present to stop sticking of the Pt spacer to the Pt sample holder. The chamber is evacuated using a rotary pump for 30 minutes and back filled three times. Experiments are run under an inert atmosphere of 99.9999% Ar passed through a further 623 K heated getter containing Cu and Mg chips.

Experimental times reported (as seen in Fig. 1) are normalized through determination of the molten oxide entering a fully liquid state, as viewed through the HT-CSLM video output. This time varied from between 5- and 25 seconds after reaching the programmed temperature of 1873 K. Reasons for this are expectedly due to variation in HT-CSLM performance, chamber maintenance, as well as effective interaction with the IR radiation due to differences in the sample surface topology (surface roughness for example^22^) and powder packing density. An example video of the FeAl-SiO_2_ system is supplied to view with article. In addition this video along with a copy of that collected at lower resolution can be found at http://www2.warwick.ac.uk/fac/sci/wmg/research/steel_processing/research/showcase.

### Micro X-ray Computer Tomography

The quenched samples were scanned using a ‘Zeiss Versa 520’ (Zeiss Microscopy; Pleasanton, USA), the machine has a 160 kV micro focus source with an alumina transmission target and charge-couple device-based detector 2000 × 2000 pixels, with a size dependent on the optic used. The machine has a cone beam X-ray source and a rotating turntable between source and detector.

The scanning parameters used were: voltage 140 kV, power 10 W, exposure 3 s, projections 3142, filter CaF_2_ 1.0mm, magnification x6.69^*^, voxel size 4.90. The additional optical focusing used was x0.4 lens, with a source-object distance of 12mm and a source-detector distance of 155mm.

The scans are then reconstructed in proprietary packages included within the systems that use filtered back projection. The 3D volume is reconstructed using a Feldkamp back projection method, creating a series of volume elements. The 3D volumes are then evaluated using ‘VGStudio MAX’ (Volume Graphics GmbH, Germany).

The surface area and volume of the iron pellet is evaluated by individual segmentation to determine the surface based on grey values, enabling selection of an appropriate isosurface. To ensure consistency the selected isosurface was automatically determined by the program through selection of sample voxels considered to be background (air) and the material (iron). Strong contrast in grey values between the oxide and metal fractions of the sample aided in the precise determination. Through this mechanism all measurements will be subject only to a consistent error across all samples.

To combat the inherent errors within XCT scanning (dependent on the given detector-source/source-object distance) a precision ball bar with a centre-to-centre distance of 5 mm is scanned. The results of the size evaluation of this can then be used to rationalize that of the experimental samples. Further information on XCT scanning parameter validation and development of the isosurface measurement can be found in previous works^3,23^.

The method by which perturbation sizes are normalized against a “quiescent” droplet surface are outlined in Supplementary Information 1.

### Phase-Field Modelling

A phase-field theory of liquid phase separation coupled to fluid flow is employed for the current study. The respective Cahn-Hillard-type and Navier-Stokes equations are solved numerically. The interfacial free energy is assumed to be γ = 600 mJ m^−2^ as given in the literature for similar conditions to the experimental approach of this work^24^. The free energies of the bulk liquid phases are taken from the regular solution model. During the simulation the temperature was fixed. The time scale of the process is set by the interplay of melt flow and chemical diffusion^25,26^. For numerical stability and efficient computation time, the time-stepping and physical properties were converted to non-dimensional quantities. The equation of motion was made dimensionless using the length and time scale ζ = 6 × 10^–6^ cm, γ = ζ^−2/D^ = 1.2 µs, where D = 3 × 10^−5^ cm^2^s^−1^ is the diffusion coefficient. The dimensionless time and spatial steps were chosen as ∆t = 1.25 × 10^−7^ and ∆x = 5 × 10^−3^. This conversion made the surface tension jump condition across the interface into an equivalent volume force to which the Navier-Stokes equations were added and solved in a numerically stable manner. Further information on the phase-field model construction is given in Supplementary Information 2.

It is well-known that the interfacial tensions can vary from 1670 mN/m to 400 mN/m depending upon the solute content of the metal and the slag chemistry^27,28^. For instance, oxygen and sulphur drastically decrease the value of interfacial tension in iron-based slag systems. In addition, an increase in the chromium content of the metal results in a decrease in the interfacial tension. FeO content of the slag also leads to a large reduction in the interfacial tension. Thus, a reasonable value of 600 mJ/m^2^ for interfacial energy was extrapolated from the experimental results in real systems^19,27–29^.

## Results and Discussion

**Figure 2:**
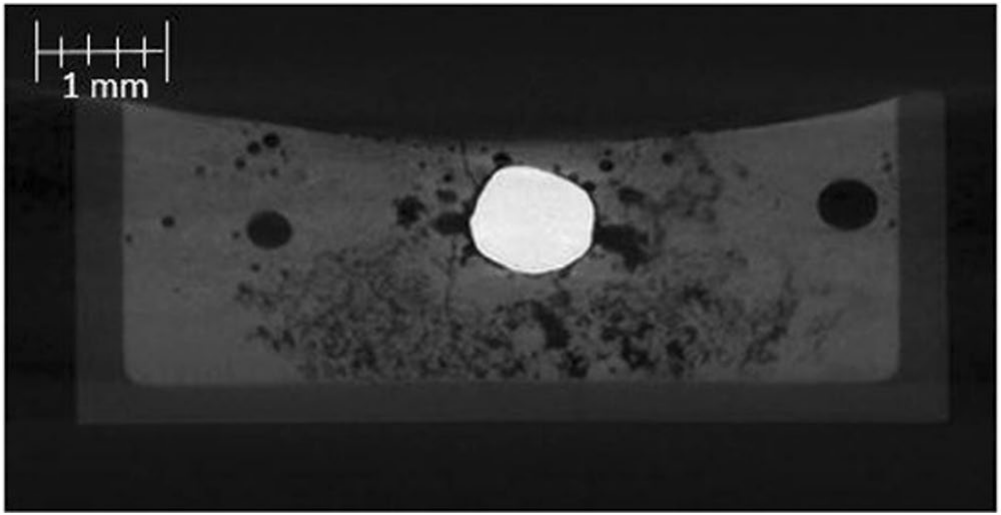
A 2D XCT reconstruction of a system at equilibrium, where the metal droplet is displayed in white, the slag in light grey, the crucible in dark grey and porosity as black (3 µm resolution scan).

**Figure 3:**
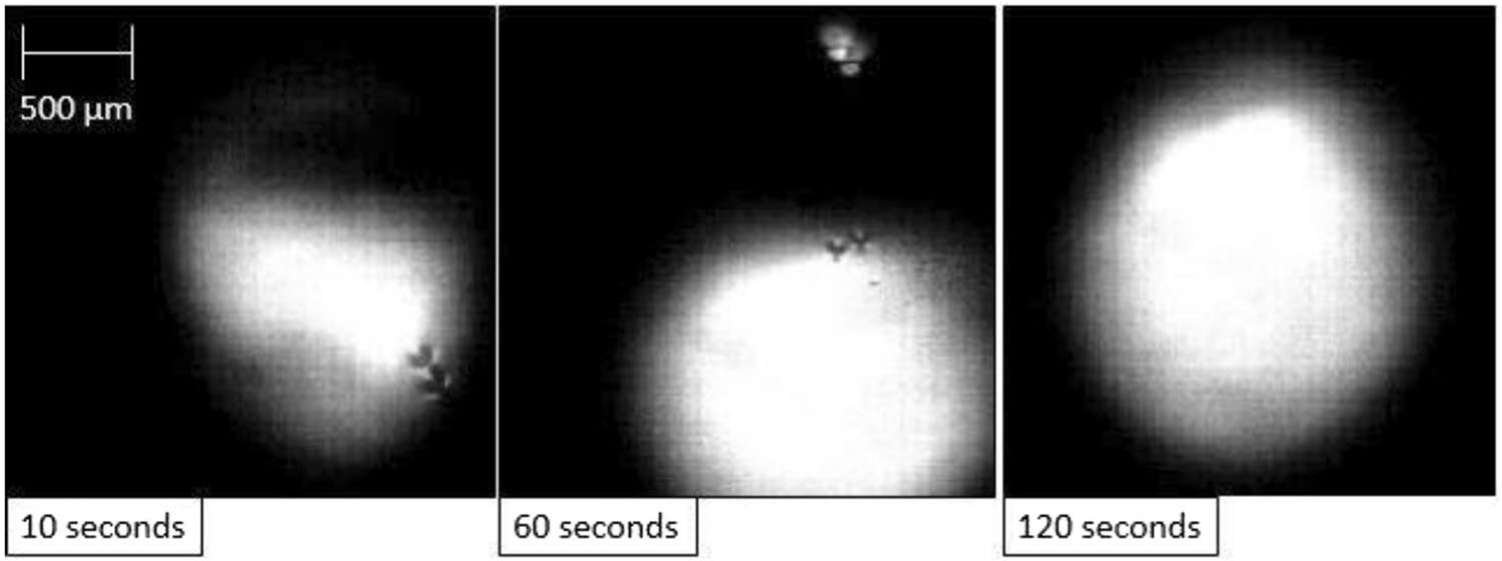
A three step sequence showing the quiescent droplet in the equilibrium system with time as imaged by the HT-CSLM through a transparent slag.

**Figure 4:**
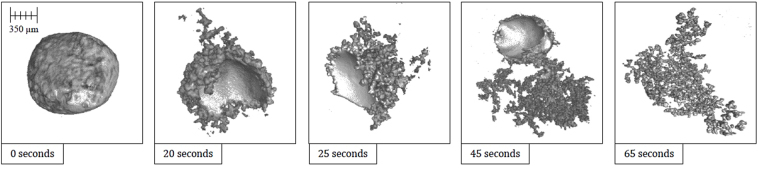
Reconstructed XCT images of the Fe-FeO system quenched at defined times; only the metal part of the sample is displayed in light grey (3 µm resolution scan).

In Fig. 2 a cross section of a quenched system at equilibrium (34 ppm O Fe droplet, with a slag containing no FeO) produced from X-ray Computed Tomography (XCT), an almost perfectly spherical geometry is found. Figure 3 is a series of stills taken from high-temperature confocal scanning laser microscopy (HT-CSLM) of the equilibrium system over a period crossing the emulsification times of both reactive systems. Very slight perturbation can be seen at times during this video, yet no individual wave shows sustained growth. Hazing and lack of distinction in the images is expected as the HT-CSLM is able to view a narrow horizontal slice of an extremely dynamic 3D system, where the imaging light is also undergoing refraction when exiting the molten surface. Both the appearance of the spherical droplet identified through XCT scanning and the lasting placidity of the droplet viewed *in-situ*, give a standard behaviour against which to set the rest of our findings. The lack of emulsification shows temperature gradients and surfactant stabilization are unable to cause SE by themselves within the systems explored^30^. XCT images presented in Fig. 4 show only the metal proportion (light grey) of scanned samples produced from the Fe-FeO system. From left to right (increasing in time after reaching 1873 K) a progression of emulsification is observed: the droplet has formed into a molten sphere on reaching temperature; perturbation begins on the droplet surface; the level of perturbation drastically increases and a small amount of material is seen to have separated; a gradually increasing portion of the droplet has entered a highly emulsified state (20–45 seconds); the entire sample has broken down to form a fully emulsified droplet (65 seconds).

**Figure 5:**
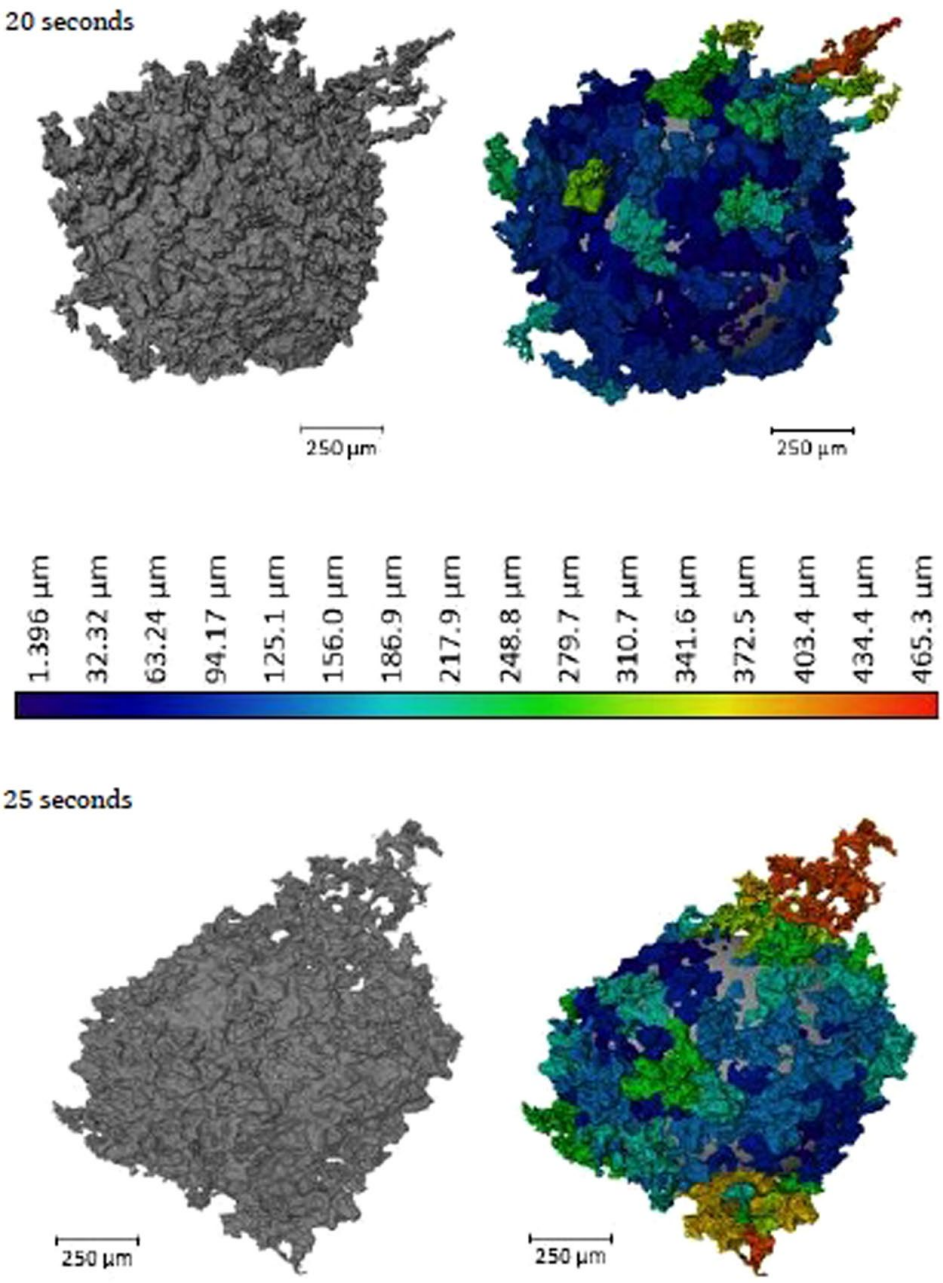
3D reconstructions of the 20- and 25-second samples, with the addition of the segmented perturbations displayed in colour dependent on length of protrusion from the segmentation sphere.

**Figure 6:**
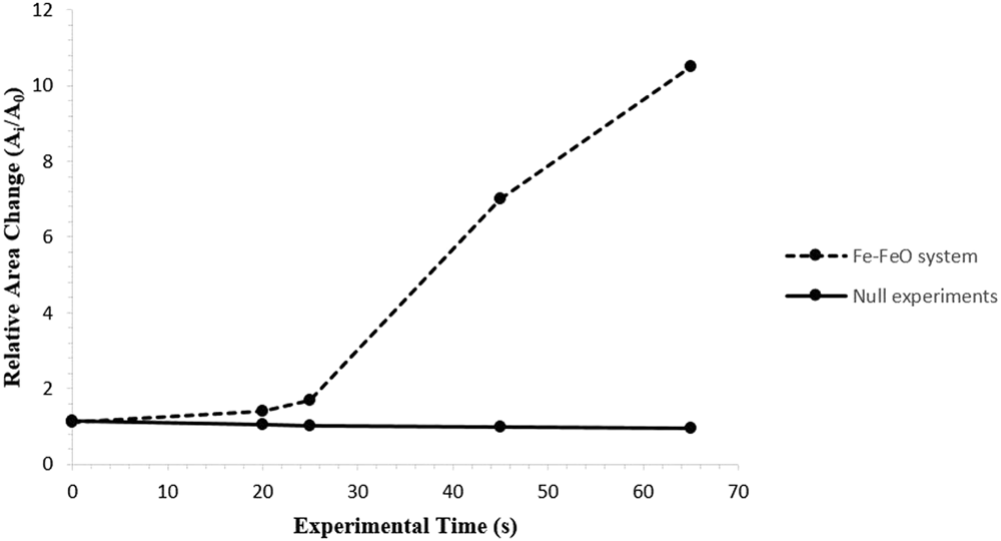
The quantified surface area of the metal droplet in the Fe-FeO system with time from XCT analysis, along with the expected surface area of the null experiment assuming a perfectly quiescent sphere; the null experiment surface area reduces with time due to slight oxidation as modelled using the equilibrium module in FactsageTM.

Figure 5 offers a qualitative view of the level of perturbation change between 20- and 25-seconds. The images at the top show the 20-second sample having a significantly higher area of the segmental perturbations in the darker blue end of the scale spectrum, as opposed to the 25-second sample where the image depicts on average a longer perturbation distance from the segmentation sphere and multiple perturbations at the very top of the length scale, above 400 µm in length. As the reaction progresses, perturbations grow due to sustained transfer of material between metal oxide and metal phase; the lowering of diffusion distances through growth allows for the faster dissipation of free energy. The effect is an increase in perturbation size and overall interfacial area increase; the energetic cost of increased interfacial area must be outweighed by the free energy release^31^. The metallic material can be envisaged as being dragged into the perturbation by the expanding surface. This is quantified in Fig. 6 where the surface areas of the metal droplets from Fig. 4 are presented along with the expected change in surface area of the null experiment previously discussed. Here it can be seen that the increased perturbation level between the 20- and 25-second samples has resulted in a slight increase in surface area. The null experiment shows a slight reduction in surface area due to loss of material through oxidation and dissolution into the slag phase with time (modelled using FactSage^TM^). Examples of heating profiles entered in the HT-CSLM for the experiments discussed are given in Fig. 6; the HT-CSLM was able to follow these profiles to within ±10 °C (range) with the most deviation at the point of change between heating to reaction temperature (1873 K) and the isotherm.

**Figure 7:**
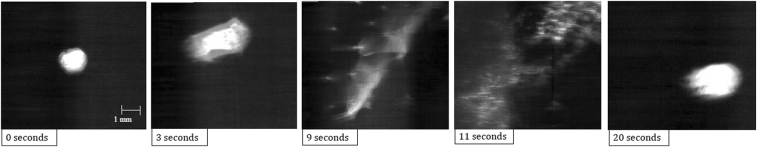
An image time step of the FeAl-SiO2 system as visualised by the HT-CSLM at lower magnification; the droplet can be seen to grow in size, spread and perturbations are observable surrounding it, followed by dispersion into a cloud covering the field of view before coalescence.

**Figure 8:**
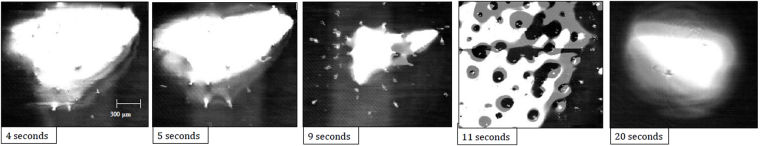
Emulsification visualization of the FeAl-SiO2 system at higher resolution via the HT-CSLM. As noted in the main text, the HT-CSLM effectively views a 2D slice of the sample; this results in perturbations from lower in the sample appearing as a halo of in-focus white line or spots around the parent droplet such as seen at 9 seconds, this is also the reason the image at 11 seconds shows a continuous metal phase, as overlapping droplets will appear as one mass.

Next the FeAl-SiO_2_ system is considered, where in Fig. 7 a time-step HT-CSLM image sequence is presented of the system during reaction at low magnification. The droplet can be seen to increase in size followed by distortion and observation of perturbations. The material then spreads into a cloud covering most of the field of view before coalescence back to a near-spherical shape. Figure 8 shows the same reaction at higher magnification; the stage of SE can clearly be observed: random perturbation beginning; growth in both size and number of these perturbations; complete breakdown/clouding of the system over the entire image; finally coalescence of the system to a single near-spherical shape. Due to the speed of this reaction the droplet was unobservable from the very commencement of slag melting, as such the first image of Fig. 8 shows slight perturbation already. Only the last image shows a smooth droplet similar to those seen in the null experiment which coincides with when the FeAl-SiO_2_ system should have reached equilibrium.

**Figure 9:**
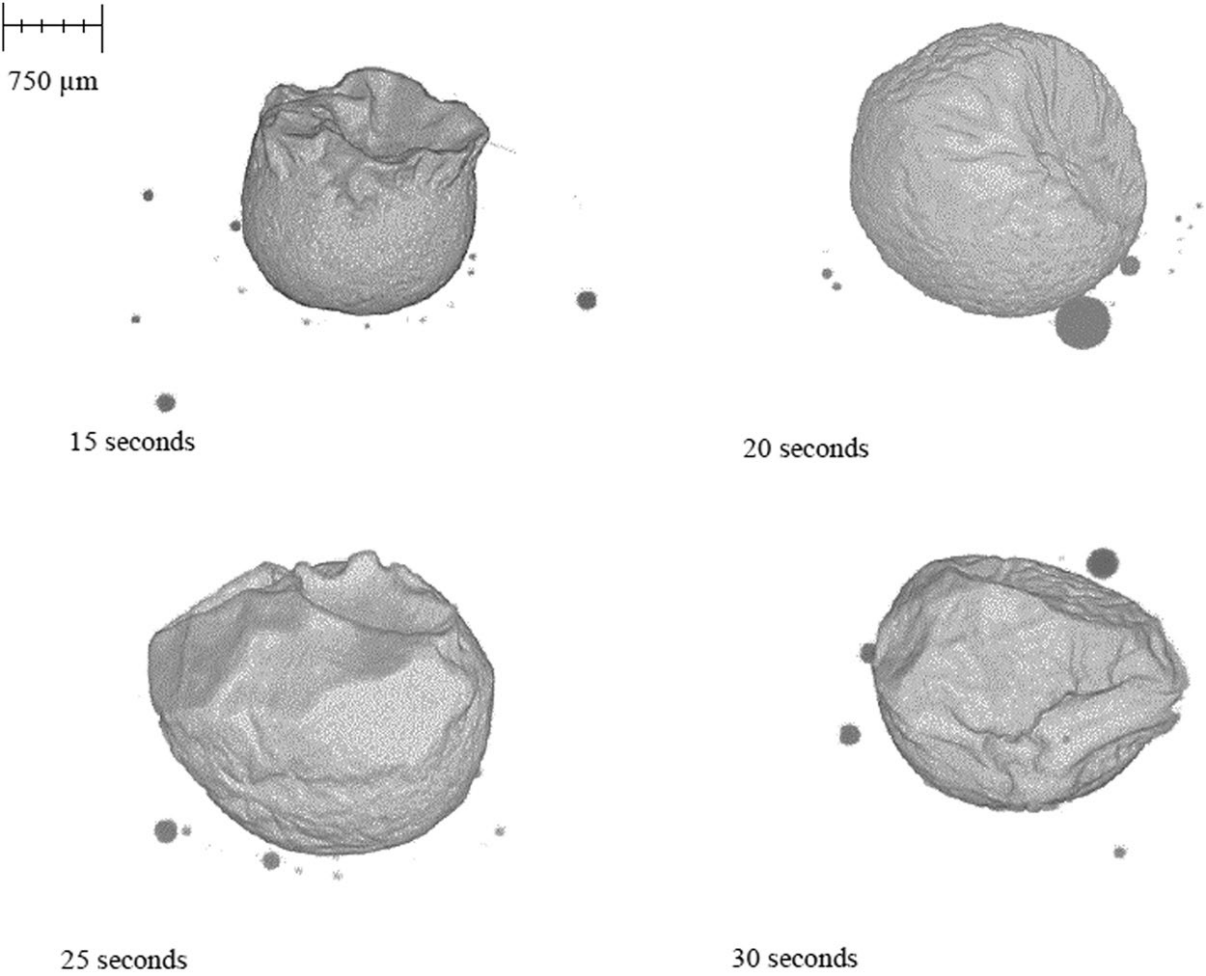
3D reconstructions of the quenched FeAl-SiO2 system where the crucible and slag phases have been removed to expose the metal phase of the sample in free space.

**Figure 10:**
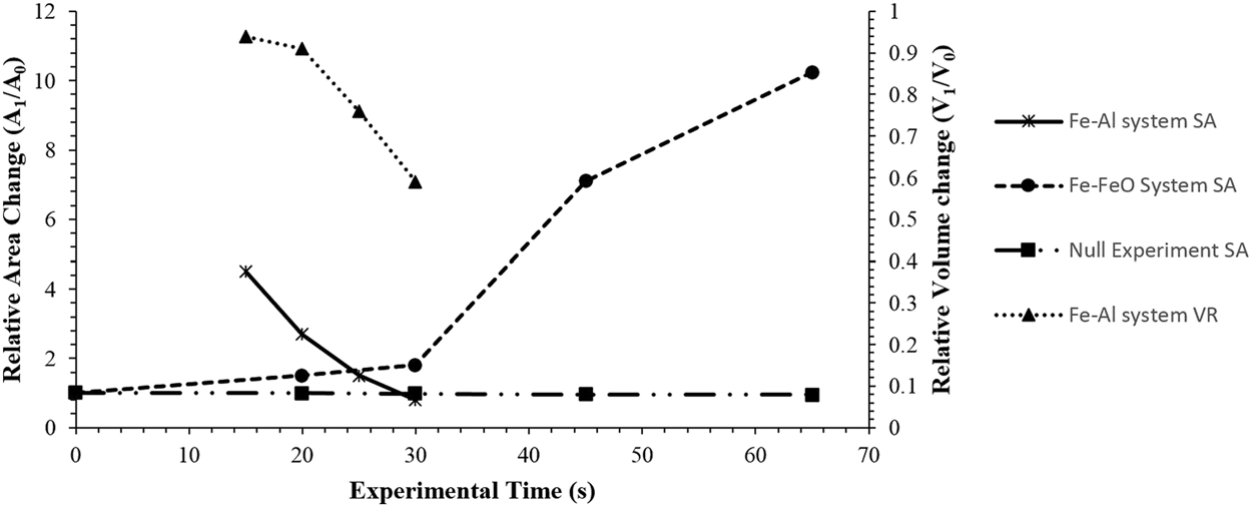
A graphical representation of the surface area change of the Fe-FeO and FeAl-SiO2 systems as measured through XCT, as well as the volume change of the Fe-Al droplet mass with time and the modelled surface area change within the null experiment.

Quenched samples of the FeAl-SiO_2_ system are displayed in Fig. 9 where the 15-second sample (the quickest time in which this sample could be captured with reduced cooling performance due to the experimental setup of this system) can be seen to have undergone significant coalescence. Yet there are still a large number of well dispersed smaller droplets throughout the sample. The 20-, 25- and 30-second samples show a reduced number and size of droplets outside the main coalesced body. The surface area and detected volume of metal in these samples has been added in Fig. 10, where it is clearly visible that the FeAl-SiO_2_ system is coalescing and near complete equilibrium within the same time frame that the Fe-FeO system begins emulsification to a significant extent. This shows significant differences in the rate of reactive species mobility in the systems, where the Fe-FeO system is most likely dependent on the rate of mass transport of FeO through the slag phase, whereas the FeAl-SiO_2_ system is likely dependent on Al mass transport in the metal phase^32,33^.

**Figure 11:**
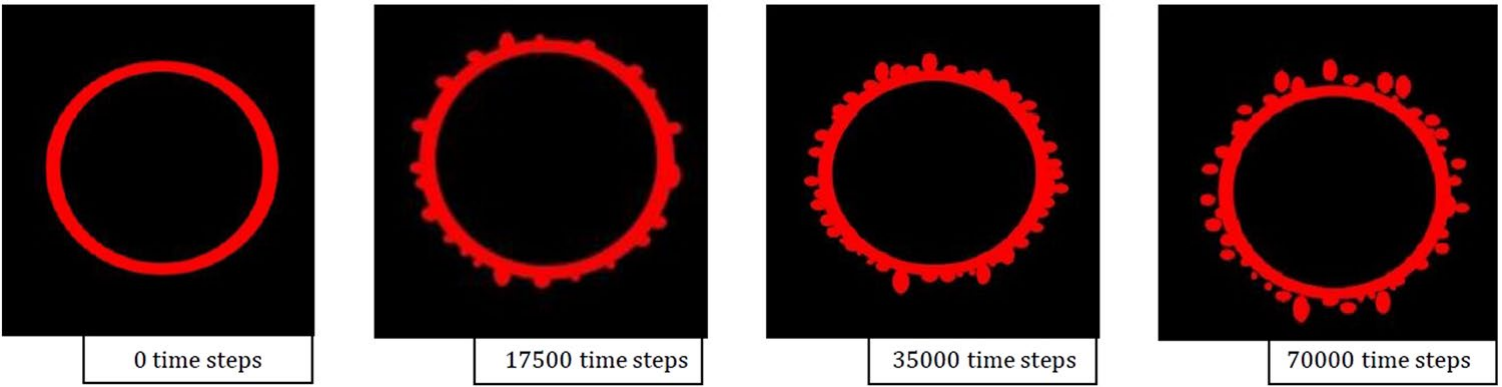
Selected graphical outputs from the 2D Fe-FeO phase-field modelling showing the progression from a quiescent sphere to a highly perturbed state where material has begun to break away from the parent droplet.

**Figure 12:**
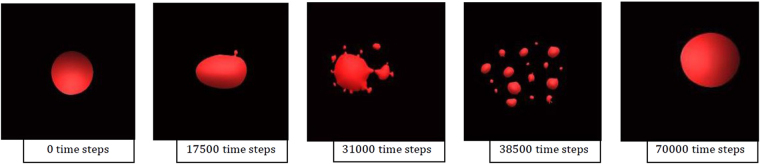
3D graphical outputs from the FeAl-SiO2 phase-field model, where the full life cycle of emulsification and coalesces can be seen within the simulated 70,000 time steps.

**Figure 13:**
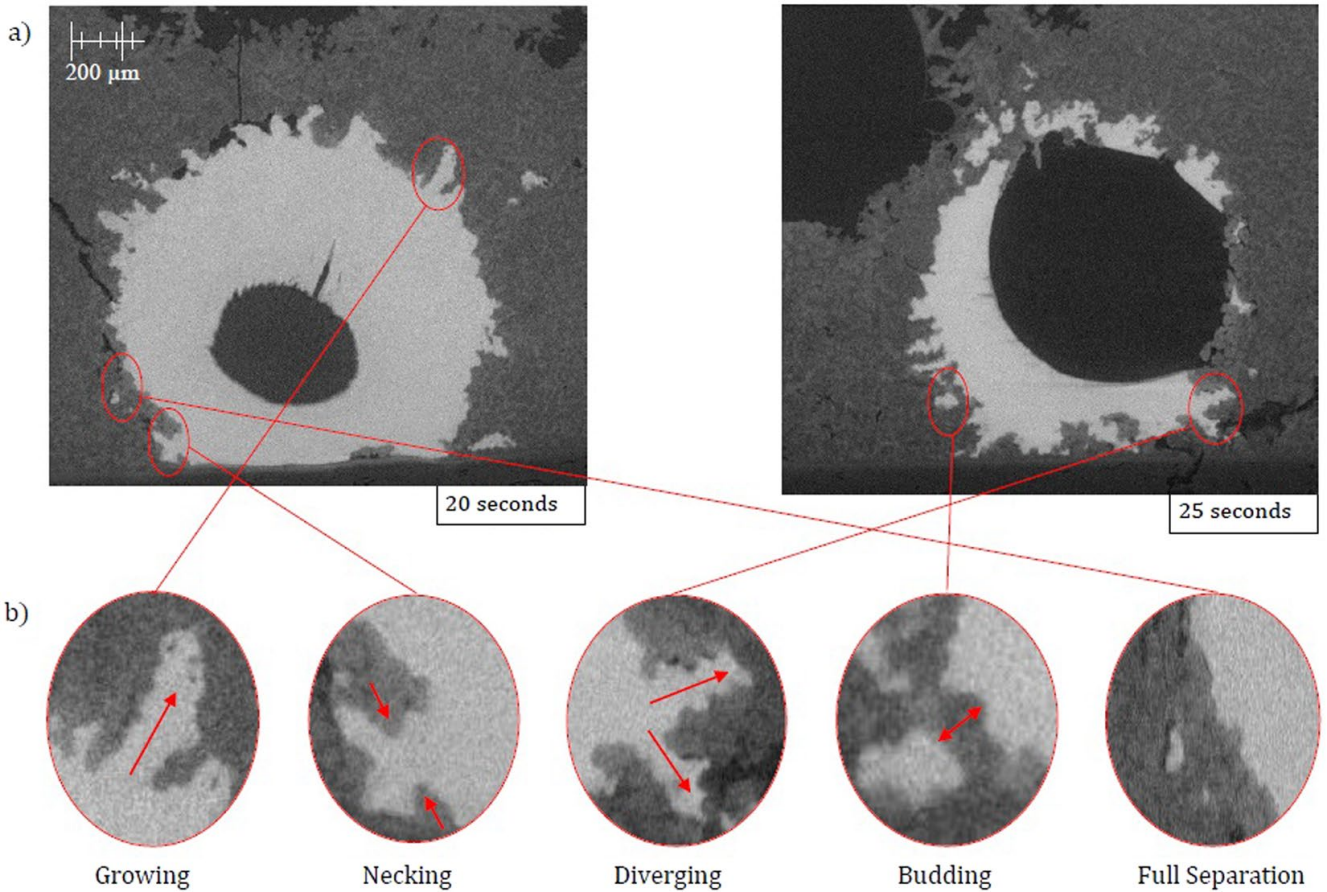
(**a**) 2D ortho slices of the XCT 20- and 25-second reconstructions where the metal phase is in light grey, slag in dark grey and porosity in black. (**b**) Magnified section of the 20- and 25-second ortho slice images where each growth cycle stage of a perturbation as identified from the phase-field modelling has been highlighted as present in the experimental samples.

The classical Fick’s law which is generally known for explaining the diffusion process, does not explain interpenetration of molecules through liquid-liquid boundaries within a horizontal capillary^8^. The grounding knowledge from these experiments is used to inform the practice of phase-field models in both 2D (for the Fe-FeO system) and 3D (for the FeAl-SiO_2_ system) with the inclusion of Navier-Stokes solutions. Figure 11 shows time-step images from the 2D Fe-FeO system where the droplet can be seen to go from the starting quiescent sphere through levels of perturbation; at t^*^ = 70,000 δt material can be seen to have necked and broken away from the parent droplet beginning the emulsification process. Figure 12 shows 3D images of the modelled FeAl-SiO_2_ system where again a similar progression in time can be seen. The model goes further than that of system 1 to view a highly emulsified state at t^*^ = 38,500 δt and culminating in a coalesced droplet at the final 70,000^th^ time step. Both models show a clear pathway of emulsification, which supports that seen in the *in-situ* HT-CSLM experiments and 3D XCT reconstructions. This defined staged pathway is further developed through the use of Fig. 13a and b where 2D ortho slices of the 20- and 25-second Fe-FeO samples are shown. Perturbations are selected in the lifecycle from left to right of the image showing stages of growth in length => necking at a position between the main droplet and perturbation tip => divergent growth of a perturbation => break away with material retraction to the main body => and finally complete separation of an offspring droplet.

**Figure 14:**
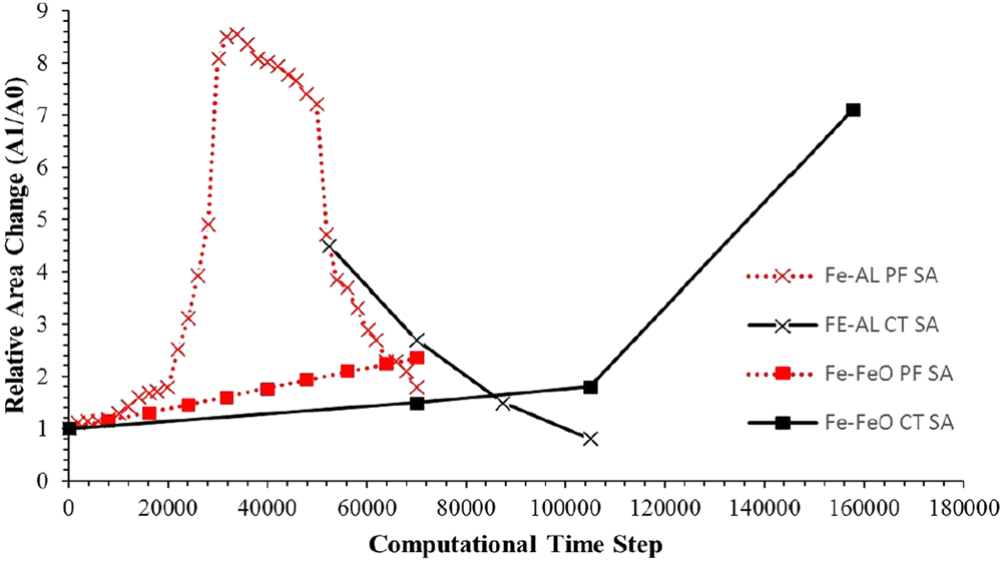
Graphical display of the transient surface area as measured via XCT for experimental samples and output geometries from the phase-field model; experimental samples have been time-step normalized through comparison of *in-situ* HT-CSLM with phase-field results.

The similarity between the patterns of behaviour observed for perturbations in both the experimental and modelling approach give strong validation of the models’ driving forces. Equally the time-step images from the 3D phase field model and the *in-situ* HT-CSLM video of the same system can then be correlated. This offers a method to deduce the number of time steps in the model equivalent to a period of time in the high-temperature experiments. This results in 3,500 time steps being equivalent to 1 second. In Fig. 14 the culmination of the two experimental systems’ interfacial area ratios where time has been converted to time steps is given, as well as the addition of both the FeAl-SiO_2_ system and Fe-FeO system interfacial area ratios computed from the phase-field model. From this it is clearly observable that both pairings have a high level of coherency between the modelled data and experimental measurement with several overlapping data points between each. Although there is slight deviation, this is expected to be due to temperature variation in the experimental method (under heating in the case of the Fe-FeO system limiting diffusion rates, and cooling during the quenching of the FeAl-SiO_2_ system reducing coalescence activity) compared to the innately held isotherm the model follows at 1873 K throughout the entire droplet reaction cycle.

**Figure 15:**
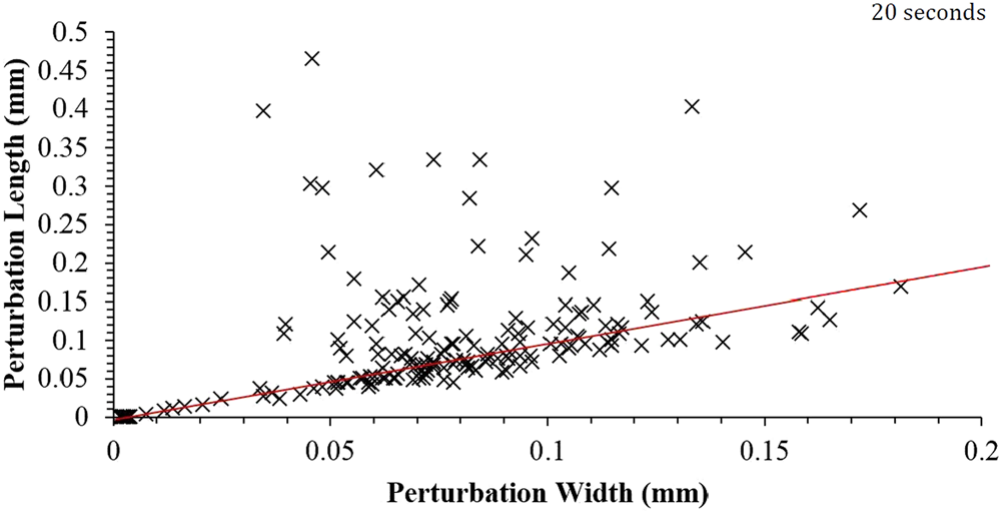
Perturbation dimensions for the 20-second Fe-FeO sample.

**Figure 16:**
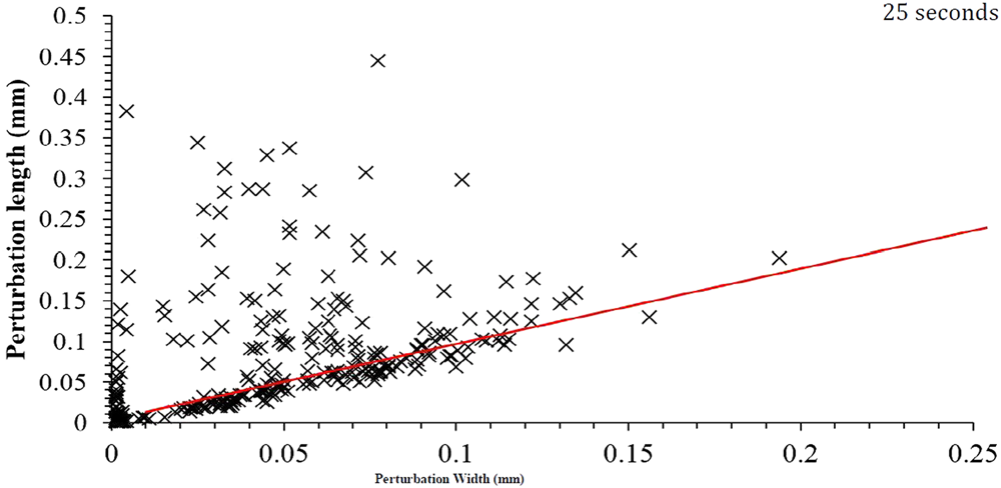
Perturbation dimensions for the 25-second Fe-FeO sample.

**Figure 17:**
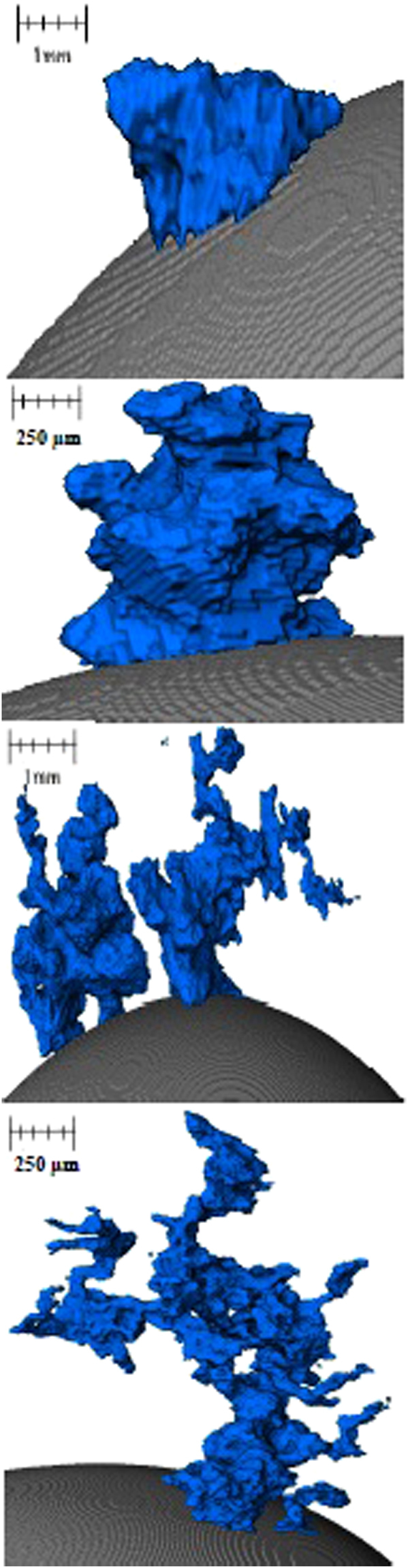
Examples of the perturbation geometries displayed in 3D space as produced via the XCT segmentation method implemented.

Figures 15 and 16 show the individual perturbation measurements for both the 20- and 25-second samples where the maximum distance from a defined quiescent sphere is taken as the length, and the narrowest point of the perturbation is taken as the width. It is visible from the redline drawn at a 1:1 aspect in each graph that a large portion of the perturbations sit on or very close to this ratio. Due to the large number of perturbations in this aspect and visual inspection of the 3D images it is reasonable to propose that the “normal” growth of a perturbation is outwards from the droplet at the same rate as they grow in width on the droplet surface. It thus follows that perturbations away from this “normal” growth regime have begun the necking phenomenon by at least 700 nm (the resolution of measurement attained within these high-resolution XCT scans). Figure 17 offers examples of segmented perturbations as a result of the method described in detail in appendix 1. These images give an appreciation for the more complex geometry’s perturbations can uptake through phenomena such as diverging growth and interaction with physical forces such as mass fluid flow due to temperature gradients or surface interaction with gas. Further to this, micro shear forces will be in effect as a result of Marangoni flow for example^34^.

**Figure 18:**
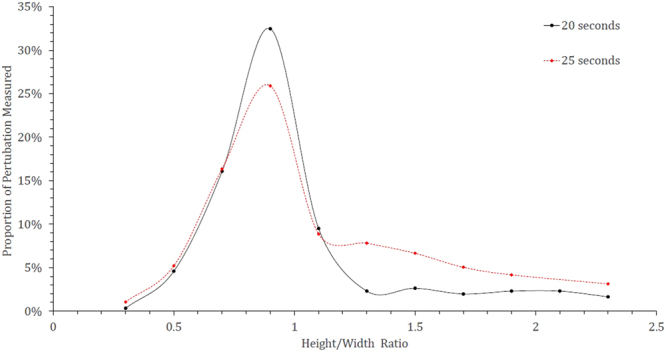
Perturbation height/width ratio distribution for the 20- and 25-second samples truncated to represent close to 85% of the total perturbations from each sample.

Figure 18 shows the distribution of perturbations in the growth cycle for both the 20- and 25-second samples where the data has been truncated to represent roughly 85% of the total perturbations measured for both samples. This equates to a maximum height/width ratio of 2.3. Both samples show the largest proportion of perturbations to have a geometry ratio of 1:1 thus quantifying the previous observation when discussing the behaviour. However, it is clear to see that the 25-second sample has a reduced portion in this “normal” growth range compared to the 20-second sample, and a greater number at higher ratios where the width is smaller than the length and thus a higher portion of the perturbations are within the necking stage of the cycle. Table 1 shows the base statistics of the perturbation measurements for both the 20- and 25-second Fe-FeO samples as well as for a random measurement of 15 perturbations from the output of the 2D Fe-FeO phase-field model. It is possible to rationalise the phase-field model scale through computation of the quiescent spherical diameter of the input 17 mg droplet, which can be coupled with the graphical output from the model at 0 time steps to offer standardization of measurement for the model’s Cartesian space output. From the table it is apparent that both the average length and width of a perturbation have increased with time, however the maximum and minimum stay within close proximity. This indicates that a perturbation may grow to a maximum length of approximately 470 µm before material breaks away from the parent droplet, as well as a maximum width and length of near 200 µm before necking begins to occur (assuming the 1:1 “normal” growth regime). Hence it is suggested that the perturbation is likely able to double in length from the time necking begins to the time material breaks away; the acceleration of perturbation growth is an interesting prospect for further investigation. It should finally be noted from the Table 1 that the manually measured sizes of perturbations present in the 70,000 time step 2D Fe-FeO modelled system are within close size proximity to those measured through XCT.

**Table 1:** The base statistics for the size of perturbations in the Fe-FeO 20- and 25-second samples as well as a random measurement of 15 perturbations from the 70,000 time-step 2D phase-field graphical depiction of the system.

	Number of Perturbations	Statistic	Width (µm)	Length (µm)	Volume (µm^3^)
20 Seconds	192 Detected	Mean Average	44.5	74.4	0.121
		Standard Dev	37.2	76.7	0.244
		Maximum	193.8	445	2.98
		Minimum	1.50	1.37	2.65 × 10^−6^
25 Seconds	305 Detected	Mean Average	69.4	91.9	0.279
		Standard Dev	40.8	81.29	0.384
		Maximum	181	465	3.08
		Minimum	1.53	1.40	2.72 × 10^−6^
70,000 Time Steps	15 Measured	Mean Average	47.0	79.0	—
		Maximum	202	457	—
		Minimum	<5	<5	—

**Figure 19:**
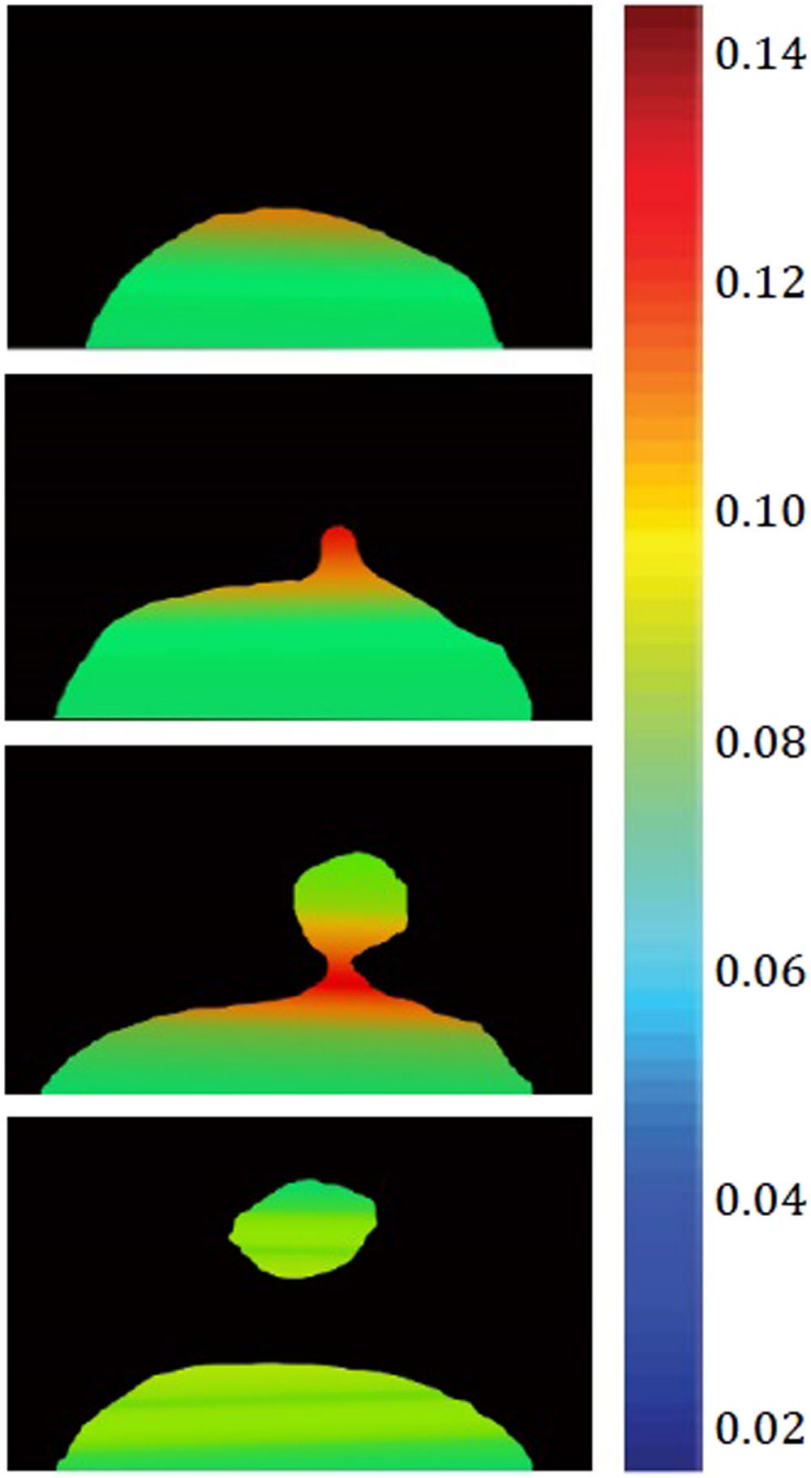
The computed distribution of surface tension through and in close proximity to a growing perturbation (t* = 15000, 20000, 30000 and 35000 Δt).

With validation of the phase-field model through physical interrogation of its graphical expression as compared to the experimental observation and measurement, the exploration of mapping both the physical force and chemical distribution in the systems is possible. The dynamics of the 3D FeAl-SiO_2_ system with respect to the stages of a perturbation’s growth and separation from the parent droplet are possible to output from the model. Figure 19 maps the interfacial tension between the two phases with respect to geographical location. The surface tension is seen to be highest at the tip of the perturbation during growth, however when the perturbation reaches a certain size this tension reduces due to reduced curvature. At the same time a shift is visible of high surface tension to the necking area of the droplet, which as the neck increases in length becomes high enough to cause release of the budding section of the perturbation, relieving the highly constrained necking area to lower the surface tension overall.

**Figure 20:**
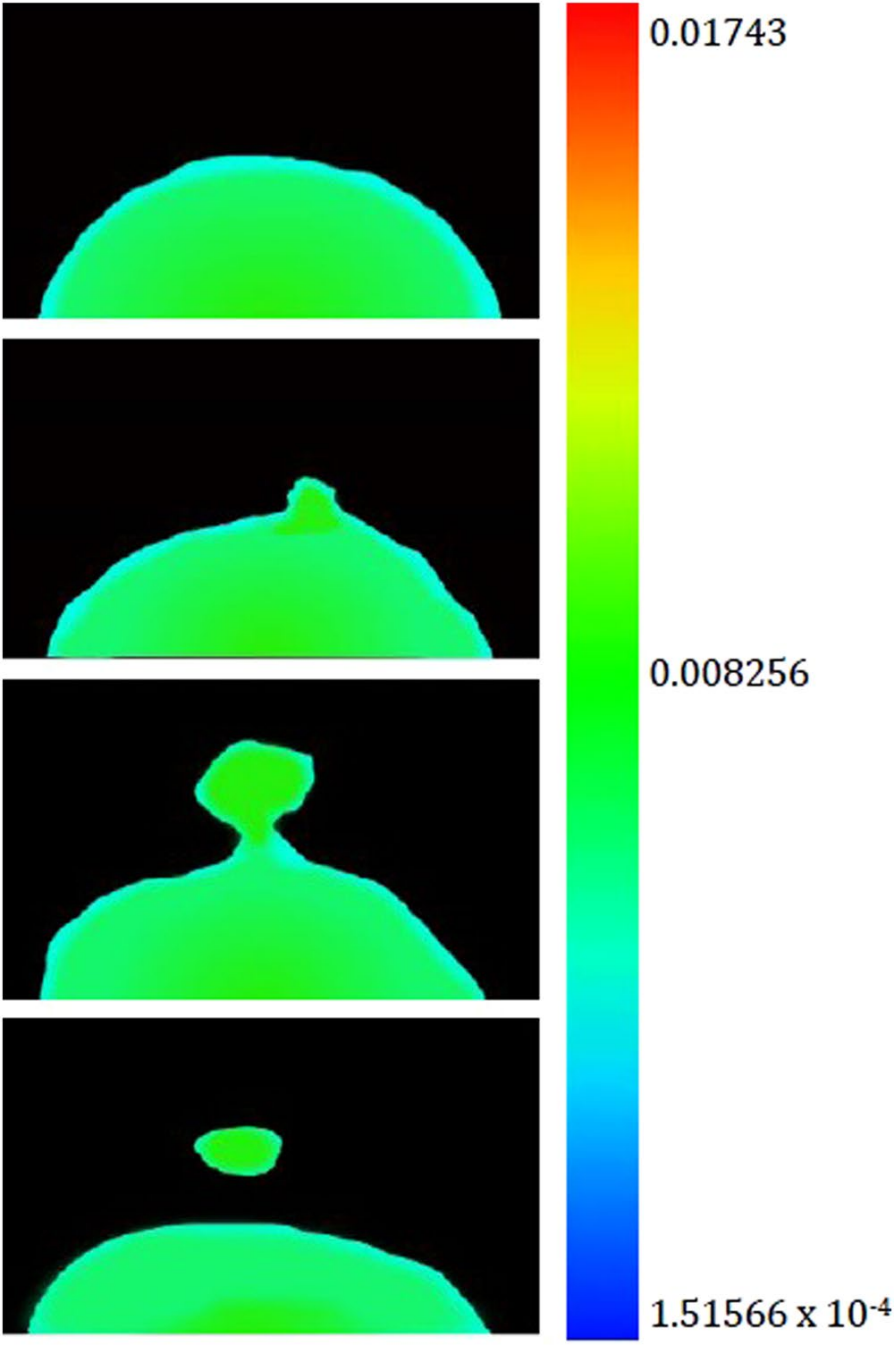
The simulated variation of chemical composition, specifically aluminium content as a distribution through the metal droplet (t* = 15000, 20000, 30000 and 35000 Δt).

**Figure 21:**
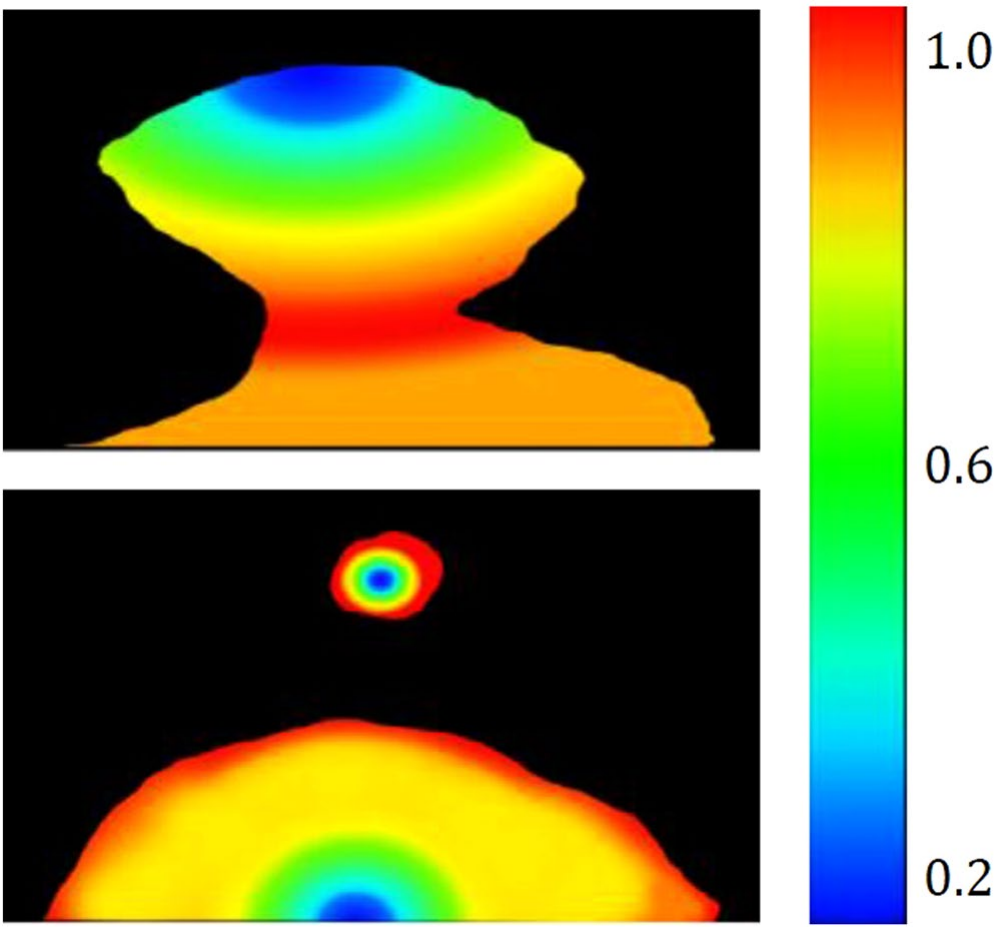
The gradient of driving force for perturbation growth and breakaway as a combination of chemical free energy and interfacial energy in close time proximity before and after material break away (t* = 20000 and 35000 Δt).

The aluminium level within the sample can be viewed as a profile of depth into the metal droplet from the slag/metal interface, as well as in the perturbation compared to the bulk droplet in Fig. 20. The model predicts a reduction in aluminium content radially outwards through the droplet, supportive of the mass transfer of aluminium being the rate-controlling step in this system. The high surface area to volume ratio within a perturbation presents a greater concentration gradient in this area of the droplet, pulling more and more aluminium from the bulk droplet into the perturbation. This is driven through the higher chemical gradient (faster than it would approach the surrounding quiescent interface) creating a higher concentration of aluminium in the “bulk” perturbation than in the surrounding surface of the droplet. Finally Fig. 21 shows the distribution of the normalized gradient of driving force including the chemical free energy and the interfacial energy within a drop. Although there is an increase in the number of reactive elements ejected into the perturbation; due to the increased curvature at the necking area, there is a larger gradient of driving force (similar to surface tension) in this area compared to the peripheral of the drop where the curvature is less as a result of the differences in the spacing between iso-concentration contours near the interface. After the separation, there is a uniform distribution of the gradient at the peripheral areas of the original drop and that of the separated droplet and both systems evolve towards decreasing their interfacial energies (surface areas). This process continues until the equilibrium state (chemical equilibrium as well as lowest possible interfacial area) is reached. It is noted that from Figs 19 to 21, the values shown in the legends are scaled and dimensionless.

Despite being able to accurately simulate the interface shape, the phase-field model is still incapable of reproducing features of the interfacial diffusion. Further development of the theory is needed to comprehensively understand the interactions involved in a liquid-liquid interface. An example would be to study whether non-isothermal effects that may result from latent heat could affect propagation of the interface. Another direction is to investigate the phenomenological relationships between diffusion flux and stress tensor. Both expressions were considered to be isotropic; however, an interface (strong gradients in concentration fields) generates anisotropy, so the relationships between thermodynamics fluxes and forces should be reconsidered.

In addition to non-isothermal effects within a given controlled system, the effect of system temperature would greatly affect the observed phenomena. Temperature is known to effect viscosity and interfacial tension as physical parameters, as well as diffusion kinetics and reaction equilibrium from a chemical view point. As such it is difficult to speculate on the true effects of temperature when competing effects may occur; for instance an increased temperature may reduce liquid viscosity of the slag phase, thus potentially encouraging faster and longer maximum perturbation growth, however at the same time causing an increase in reactant mobility (SiO_2_ in the slag phase for example) resulting in a change of the reactions rate limiting step with a possibility to reduce or completely remove the driving force for spontaneous emulsification to occur. The authors are keen to investigate these effects and the reliance on temperature among other parameters such as concentration of reactant or the introduction of an inhibiting species (sulfur for example) in the future.

## Conclusion

The coupling of experimental techniques and modelling has allowed the in-depth investigation into the pathway of not only SE in a two-phase system, but also the life cycle of a perturbation and the limits of size before material breaks away to form the emulsion.

Specifically quantified and theoretical novel findings from the experimental investigation include:

Using the Fe-FeO system:

1. A 1:1 normal height/width growth ratio for perturbation before necking occurs.
2. A maximum directional growth of 200 µm before necking occurs.
3. A maximum growth in length of up to 470 µm before material breakaway from the perturbation head (budding).
4. A clear shift in the population distribution of perturbations within their “life-cycle” to a greater proportion having entered the process of necking/post necking between the 20- and 25 second sample, thus indicating the progression towards emulsification during this time period.
  Using the FeAl-SiO_2_ system:
5. The use of both low and high magnification to track the spontaneous emulsification of the model presents a new insight into how the clouding of the emulsified state occurs as well as the growth of perturbations both in size and number over the initial stages of reaction.
6. The 3D visualizations shows the coalescence of significant portions of the metal droplet to form a large droplet again as the vigour of the reaction decreases. However although a large droplet is formed small droplets still exist. This coalescence model is similar and well explained by DVLO theory, which considers particle attraction, interfacial angle penalties and film drain barriers^35^. This observation was previously discussed by the authors as a possibility, however the new evidence offers clearer adherence to the phenomenon^3^.

The *in-situ* observation of emulsification greatly backs spontaneous emulsification being perturbation growth led. The ability to track these phenomena in ultra-high-temperature systems such as those presented is not only of significant importance to directly related fields such as liquid steel refining of impurity removal such as phosphorus, manganese or silicon. The converse is also true when considering the ability to predict stable new alloy compositions with respect to interaction with ladle slags and mould fluxes (molten oxide phases). In these reactions knowledge and ability to track the interfacial area between reacting liquid phases offers the potential for precise control in macroscopic systems where dispersions of droplets can have significant control over the observed performance. The direct application of the systems presented here are refining in basic oxygen steelmaking controlled by emulsion kinetics^36,37^ and the composition control and stability of lightweight high-aluminium steel grades.

The formation of a physically validated phase-field model to interrogate SE systems is of great wider value. In addition to the brief summary at the beginning of this article, advanced forms of controllable micro dispersions are under use in the fields of medicine^38^, polymeric nanoparticle production^39^ and efficient recycling of metals^40^. This system is highly dynamic and difficult to capture reliable first principle data for, due to the extreme temperatures involved. As a result, reactions and movement can be magnitudes greater than those seen at room temperature. A greater number of time-step iterations would expectedly be needed for the model to produce a complete life cycle of an emulsifying system in some of the slower reacting systems. Although a 3D model of a system is clearer for qualitative observation and to achieve greater absolute accuracy, the findings in this work show a 2D phase-field system to have strong experimental agreement, and offer a viable option when computation time is considered to track the physical phenomena present in highly dynamic systems over a greater period of time and the level of accuracy possible to acquire for these systems to date presents a viable data source for the prediction of SE occurrence. It is however thought the true engineering of geometries through the process would require more accurate data and development of the model used here.

## Electronic supplementary material

In-situ video of spontaneous emulsification

Supplementary information

## References

[1] López-Montilla, J. Spontaneous emulsification: mechanisms, physicochemical aspects, modeling, and applications. *J. Dispers*. at http://www.tandfonline.com/doi/pdf/10.1080/01932690208984202 (2002).

[2] López-Montilla JC, Herrera-Morales PE, Shah DO (2002). New method to quantitatively determine the spontaneity of the emulsification process. Langmuir.

[3] Assis, A. N. *et al*. Spontaneous Emulsification of a Metal Drop Immersed in Slag Due to Dephosphorization: Surface Area Quantification. *Metall. Mater. Trans. B* 568–576 10.1007/s11663-014-0248-z (2014).

[4] Hartung HA, Rice OK (1955). Some Studies of Spontaneous Emulsification. J. Colloid Sci..

[5] S. Sahin O, Bliznyuk ARCKS (2016). 2016 Microfluidic EDGE emulsification, the importance of interface interaction on droplet formation and pressure stability. Sci. reports Nat..

[6] Costa e Silva A (2012). Estimating Viscosities in Iron and Steelmaking Slags in the CaO-Al2O3-MgO-SiO2-(TiO2) System with Basis on a Thermodynamic Model. J. Mater. Res. Technol..

[7] Waarden, M. Van Der. The process of spontaneouse emulsification. *Koninklijke/Shell-Laboratorium* 140–150 (1952).

[8] Solans C, Morales D, Homs M (2016). Spontaneous emulsification. Curr. Opin. Colloid Interface Sci..

[9] Nishimi T, Miller CA (2001). Spontaneous Emulsification Produced by Chemical Reactions. J. Colloid Interface Sci..

[10] Lewis JB, Pratt HRC (1953). Oscillating Droplets. Nature.

[11] Garner FH, Nutt CW, Mohtadi MF (1955). Pulsation and Mass Transfer of Pendent Liquid Droplets. Nature.

[12] Haydon DA (1955). Oscillating Droplets and Spontaneous Emulsification. Nature.

[13] Ganachaud F, Katz JL (2005). Nanoparticles and nanocapsules created using the ouzo effect: Spontaneous emulsification as an alternative to ultrasonic and high-shear devices. ChemPhysChem.

[14] Minehan WT, Messing GL (1992). Synthesis of spherical silica particles by spontaneous emulsification. Colloids and Surfaces.

[15] Wang D (2000). A novel method for controlling the surface morphology of polymeric membranes. J. Memb. Sci..

[16] Komaiko J, McClements DJ (2015). Low-energy formation of edible nanoemulsions by spontaneous emulsification: Factors influencing particle size. J. Food Eng..

[17] Mohammed T, Stephen Spooner SS (2014). Phosphorus: The Noose of Sustainability and Renewability in Steelmaking. JOM.

[18] Bouchemal K, Briançon S, Perrier E, Fessi H (2004). Nano-emulsion formulation using spontaneous emulsification: Solvent, oil and surfactant optimisation. Int. J. Pharm..

[19] Gaye H, Lucas LD, Olette M, Riboud PV (1984). Metal-Slag Interfacial Properties: Equilibrium Values and ‘Dynamic’ Phenomena. Can. Metall. Q..

[20] López-Montilla JC, Herrera-Morales PE, Pandey S, Shah DO (2002). Spontaneous Emulsification: Mechanisms, Physicochemical Aspects, Modeling, and Applications. J. Dispers. Sci. Technol..

[21] Stephen Spooner, Z. L. and S. S. Spontaneouse Emulsification as a Function of Material Exchange. *Sci. reports Nat*. **Accepted** (2017). 10.1038/s41598-017-05861-5 PMC5511232 28710442

[22] Auinger M, Ebbinghaus P, Blumich A (2014). A. E. Effect of Surface Roughness on optical heating of metals. J. Eur. Opt. Soc. Rapid Publ..

[23] Kumar, J., Attridge, A., Wood, P. K. C. & Williams, M. A. Analysis of the effect of cone-beam geometry and test object configuration on the measurement accuracy of a computed tomography scanner used for dimensional measurement, 10.1088/0957-0233/22/3/035105 (2011).

[24] Hoyer W, Kaban I, Merkwitz M (2003). Liquid-liquid interfacial tension in immiscible binary Al-based alloys. J. Optoelectron. Adv. Mater..

[25] Warren JA, Boettinger WJ (1995). Prediction of dendritic growth and microsegregation patterns in a binary alloy using the phase-field method. Acta Metall. Mater..

[26] Gránásy L (2003). Growth of ‘dizzy dendrites’ in a random field of foreign particles. Nat. Mater..

[27] Jimbo, I. and Cramb, A. W. In Proceedings of the Sixth International Iron and Steel Congress, The Iron and Steel Institute of Japan 499–504 (1990).

[28] Cramb, A. W., Chung, Y., Harman, J., Sharan, A. and Jimbo, I. The slag/metal interface and associated phenomena. In *Proceedings of Molten Slags, Fluxes and Salts ’97 Conference* 35–50 (1997).

[29] Kozakevich, P. and Urbain, G. R. Surface tension of liquid iron and its alloys. *Mem. SCi. Rev. Met*. 717–34 (1961).

[30] Spooner, S. *et al*. Investigation into the Cause of Spontaneous Emulsification of a Free Steel Droplet; Validation of the Chemical Exchange Pathway. *Metall. Mater. Trans. B*, 10.1007/s11663-016-0700-3 (2016).

[31] Muhmood L, Viswanathan NN, Seetharaman S (2011). Some Investigations into the Dynamic Mass Transfer at the Slag–Metal Interface Using Sulfur: Concept of Interfacial Velocity. Metall. Mater. Trans. B.

[32] Sundman B (1991). An assessment of the Fe-O system. J. Phase Equilibria.

[33] Selleby M (1997). An Assessment of the Ca-Fe-O-Si System. Metall. Mater. Trans. B.

[34] Chung Y, Cramb A (2000). Dynamic and equilibrium interfacial phenomena in liquid steel-slag systems. Metall. Mater. Trans. B.

[35] Bhattacharjee S, Elimelech M, Borkovec M (1998). DLVO Interaction between Colloidal Particles: Beyond Derjaguin’s Approximation. Croat. Chem. Acta.

[36] Meyer, H. W., Porter, W. F., Smith, G. C. & Szekely, J. Slag-Metal Emulsions and their Importance in {BOF} Steelmaking. *Journal of Metals* 35–42 (1968).

[37] He Q, Standish N (1990). A model study of residence time of metal droplets in the slag in BOF steelmaking. ISIJ Int..

[38] Saberi AH, Fang Y, McClements DJ (2013). Effect of glycerol on formation, stability, and properties of vitamin-E enriched nanoemulsions produced using spontaneous emulsification. J. Colloid Interface Sci..

[39] Quintanar-Guerrero D, Allémann E, Doelker E, Fessi H (1997). A mechanistic study of the formation of polymer nanoparticles by the emulsification-diffusion technique. Colloid Polym. Sci..

[40] Ye J, Sahai Y (1996). Interaction and interfacial tension between aluminum alloys and molten salts. Materials transactions-JIM.

