# Supplementary material for: Quantifying the Pathway and Predicting Spontaneous Emulsification during Material Exchange in a Two Phase Liquid System
Source: Sci Rep. 2017 Oct 30;7:14384. doi: 10.1038/s41598-017-14638-9 (PMC5662617; doi:10.1038/s41598-017-14638-9)
Supplement: Supplementary file 2 — Supplementary information [file 41598_2017_14638_MOESM2_ESM.pdf]

Quantifying the Pathway and Predicting Spontaneous Emulsification during Material Exchange in a Two Phase Liquid System

Stephen Spooner*, Alireza Rahnama, Jason M. Warnett, Mark A. Williams, Zushu Li & Seetharaman Sridhar

WMG, University of Warwick, Coventry, UK, CV4 7AL

* Corresponding email address: s.spooner@warwick.ac.uk

Supplementary Information

1. Method of determining perturbation length

In order to measure the perturbation length we have to define a “quiescent” surface location at which to measure from as well as define the separation of the surface into distinct non-overlapping perturbations. To do this an effective removal of the inner droplet is carried out in the XCT analysis software, followed by a systematic measurement from a set point to the tip of each perturbation.

The first stage is to define an average minimum of the wells between raised points on the droplet surface (the bottom of the perturbations). A sphere whose edge overlaps the average minimum is then created, such as figure 1.

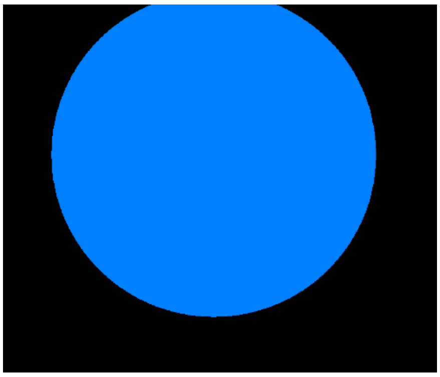

Figure 1 An example of the sphere created from the minima averaging of perturbations

This sphere is then subtracted from the original droplet geometry, leaving only the surface of the droplet; the layer which is made due to perturbation of the system. Figure 2a shows a 2D slice of the 20-second sample, and figure 2b shows the residual “surface” layer of the droplet after removal of the internal droplet.

a) b)

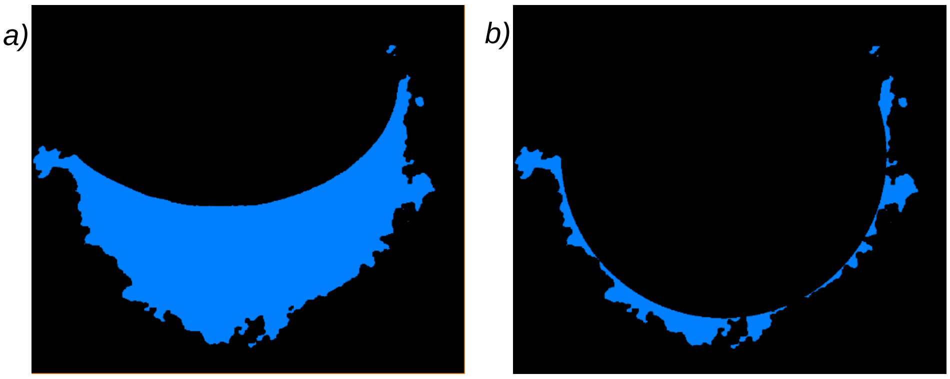

Figure 2 a) A 2D slice of the 20-second sample, where the metal has been highlighted in blue and all other material removed. b) The resultant volume left after removal of the interior of the droplet through overlapping of the minima averaging sphere.

We then use the software to automate a segmentation of the remaining surface layer via splitting of the volume between the originally identified perturbation minima. This results in segmentation of the 2D slice in this example as seen in figure 3. As visiblee from figure 3, the sofware was not completely able to segment every perturbation, mainly due to the phenomena depicted in the main papers figure 13 "diverging growth". However this element of error was consistent across both the 20- and 25-second samples, as was unavoidable in order to standardize and replicate the measurment for the number of perturbations present in the systems.

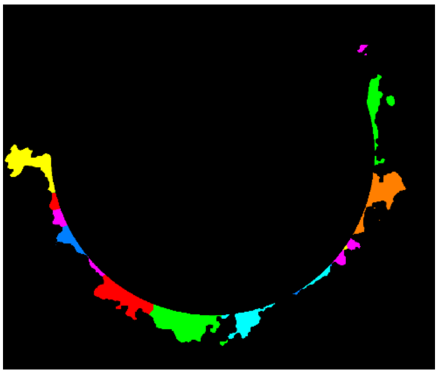

Figure 3 The effective segmentation of the droplet surface as a result of the automated method within the software splitting at defined minima. Each colour represents a separately defined perturbation.

Grey-scale values are then assigned to each voxel within the reconstruction with regards to their radial distribution from the centre of the sphere (figure 1) which was removed from the original droplet. Where all voxels under the original sphere location are assigned as pure black, followed by a linear function of 28-point grey-scale distribution over the rest of the image as seen in figure 4.

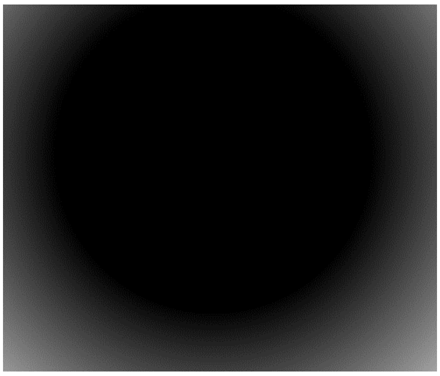

Figure 4 The grey-scale distribution of the sample space as defined from the original average minima produced sphere size. The black circle is in the space of the sphere and the grey values lighten radially outwards from its surface.

Overlapping the radial grey scale and the segmented surface volume then allows us to calculate the outer most point of each perturbation by defining its grey-scale value which is directly related to its distance from the perturbation minima.

2. Phase-Field Simulation

To model the droplet behaviour in the experimental emulsification systems, the dual effects of non-Newtonian rheology and the moving interface had to be considered.

Navier-Stokes equations were employed to model the unsteady, viscous, incompressible and immiscible two-fluid systems in two- and three-dimensional space:

$$\rho_i\left(\frac{\delta u_i}{\delta t}+u_i.\nabla u_i\right)=-\nabla p_i+\nabla.\left[\eta_i\left(\nabla u_i+\nabla u_i^T\right)\right]+\boldsymbol{SF} \qquad \text{in } \Omega_i \qquad (1)$$

and

$$\nabla.u_i=0 \qquad \text{in } \Omega_i \qquad (2)$$

where $\rho_i(x,t)$ refers to the density, $\rho_i(x,t)$ refers to the density, $u_i(X,t) = (u_1(x,t),u_2(x,t),u_3(x,t))$ denotes velocity, $p_i(X,t)$ and $\eta_i(X,t)$ are the pressure and viscosity of fluids $i=1,2$, respectively. The transcript T refers to transpose and Ω represents the domain of each fluid. ***SF*** represents surface tension force. Normally this surface tension is considered a singular force and defined as $\boldsymbol{SF}_{sing}=-\sigma\kappa_\Gamma n$ where σ is the surface tension coefficient and κ is the mean curvature of the interface[1] and δ_Γ is the surface delta function[2]. In order to make the mathematical definition of surface tension compatible with the phase-field formalism, a regular ***SF*** force was employed. It is then possible to implement the Laplace-Young calculation[3] at the exact interface boundary using a continuum surface force. This converts the surface tension jump condition across the interface into an equivalent volume force to which the Navier-Stokes equations are added. Thus ***SF*** is defined as:

$$SF=6\sqrt{2}\varepsilon\sigma\nabla.\left(|\nabla c|^2 I-\nabla c\otimes\nabla c\right) \qquad (3)$$

where ε is the gradient coefficient, c is the composition, I is the identity matrix and $(\nabla c\otimes\nabla c)_{ij}=\frac{\delta c}{\delta x_i}\frac{\delta c}{\delta x_j}$ is the usual tensor product.

Density and viscosity are defined as linear functions of the phase-field[4] and harmonic interpolation and linear interpolation for the density and viscosity are used respectively:

$$\frac{1}{\rho(\phi)}=\frac{1+\phi}{2\rho_1}+\frac{1-\phi}{2\rho_2} \qquad (4a)$$

$$\eta(\phi)=\frac{1+\phi}{2}\eta_1+\frac{1-\phi}{2}\eta_2 \qquad (4b)$$

where ϕ is the phase-field variable and is defined as $\phi=\frac{m_1-m_2}{m_1+m_2}$,where m_1 and m_2 refer to the masses of fluids 1 and 2. Harmonic interpolation is employed because the solution of the Cahn-Hilliard equation does not satisfy the maximal principle[2]. Linear interpolation cannot be bound away from zero, while the harmonic interpolation results in the desired properties due to the L∞-bound of the solution[3]. An advective Cahn-Hilliard equation is introduced as the governing equation for the phase-field:

$$\phi+u.\nabla\phi=\nabla.\left(M(\phi)\nabla\mu\right) \qquad (5a)$$

$$\mu=F'(\phi)-\varepsilon^2\Delta\phi \qquad (5b)$$

where μ is the bulk velocity and $M(\phi) =1-\phi 2$ denotes the phase-field non-negative mobility[4]. $F(\phi)$ is the double well potential of a unit volume of homogenous material of composition ϕ. To derive the

Cahn-Hilliard equation with variable mobility the chemical potential \$\mu\$ is added as the vibrational derivative of Ginzburg-Landau free energy, $\mu := \delta G/\delta\phi = F'(\phi) - \varepsilon^2 \Delta\phi$ which defines the flux as $_T := -M(\phi)\nabla\mu$. As a result of mass conservation $\delta\phi/\delta t = -\nabla ._T$. The natural and no-flux boundary conditions used are:

$$\frac{\delta\phi}{\delta n} = \nabla\phi . n = 0 \qquad \text{and} \tag{6a}$$

$$_T . n = 0 \qquad \text{on } \delta\Omega \tag{6b}$$

where n is a unit normal vector to $\delta\Omega$. The parameters used in this study were taken from the literature[5–7]. A semi-Implicit-Fourier-Spectral-Method[8] is then used for numerical analysis with a periodical boundary condition. The system size for the simulation is 300Δx X 300Δx X 300Δx for the 2D and 1000Δx X 1000Δx X 1000Δx for the 3D simulation. The method is programmed using C++ and the output is visualized using in-house visualization software (ARVisual) developed by our research group.

References

1. Assis, A. N. *et al.* Spontaneous Emulsification of a Metal Drop Immersed in Slag Due to Dephosphorization: Surface Area Quantification. *Metall. Mater. Trans. B* 568–576 (2014). doi:10.1007/s11663-014-0248-z
2. Liu, C. & Shen, J. A phase field model for the mixture of two incompressible fluids and its approximation by a Fourier-spectral method. *Phys. D Nonlinear Phenom.* **179,** 211–228 (2003).
3. Caffarelli, L. A. & Muler, N. E. An L∞ bound for solutions of the Cahn-Hilliard equation. *Arch. Ration. Mech. Anal.* **133,** 129–144 (1995).
4. Badalassi, V. E. *et al.* Computation of multiphase systems with phase field models. *J. Comput. Phys.* **190,** 371–397 (2003).
5. Costa e Silva, A. Estimating Viscosities in Iron and Steelmaking Slags in the CaO-Al2O3-MgO-SiO2-(TiO2) System with Basis on a Thermodynamic Model. *J. Mater. Res. Technol.* **1,** 154–160 (2012).
6. Selleby, M. An Assessment of the Ca-Fe-O-Si System. *Metall. Mater. Trans. B* **28,** 577–596 (1997).
7. Sundman, B. An assessment of the Fe-O system. *J. Phase Equilibria* **12,** 127–140 (1991).
8. Chen, L. Q. & Shen, J. Application of semi-implicit Fourier-spectral method to phase field equations. *Computer Physics Communication* **108,** 147–158 (1998).
